# Vaccination of rabbits with immunodominant antigens from *Sarcoptes scabiei* induced high levels of humoral responses and pro-inflammatory cytokines but confers limited protection

**DOI:** 10.1186/s13071-016-1717-9

**Published:** 2016-08-08

**Authors:** Rosa Casais, Victor Granda, Ana Balseiro, Ana del Cerro, Kevin P. Dalton, Roxana González, Pablo Bravo, J. M. Prieto, Maria Montoya

**Affiliations:** 1 Servicio Regional de Investigación y Desarrollo Agroalimentario (SERIDA), Centro de, Biotecnología Animal, La Olla-Deva, 33394 Asturias, Spain; 2 Servicio Regional de Investigación y Desarrollo Agroalimentario (SERIDA), Finca experimental La Mata, Programa de Investigación Forestal (PIF). Área de Cultivos Hortofrutícolas y Forestales, La Mata s/n, 33825 Asturias, Spain; 3 Instituto Universitario de Biotecnología de Asturias, Departamento de Bioquímica y Biología Molecular, Edificio Santiago Gascón, Campus El Cristo, Universidad de Oviedo, 33006 Oviedo, Spain; 4 Clinical Research Centre (CRC), Barts Health NHS Trust, 2 Newark Street, Abernethy Building, Whitechapel, London, UK; 5 Centre de Recerca en Sanitat Animal (CReSA), UAB-IRTA, Campus de la Universitat Autònoma de Barcelona, Bellaterra Cerdanyola del Vallès, Spain; 6 The Pirbright Institute, Ash Road, Pirbright, Woking, Surrey, UK

**Keywords:** Sarcoptic mange, *Sarcoptes scabiei*, Immunodominant antigens, Vaccine candidates, Clinical protection

## Abstract

**Background:**

Vaccination is an attractive ecological alternative to the use of acaricides for parasite control. However, effective anti-parasite vaccines against sarcoptic mange have not yet been developed. The purpose of this study was first to identify *Sarcoptes scabiei* immunodominant antigens and second to evaluate them as vaccine candidates in a rabbit/*S. scabiei* var. *cuniculi* model.

**Methods:**

The *S. scabiei* Ssλ15 immunodominant antigen was selected by immunoscreening of a *S. scabiei* var. *hominis* cDNA. The full-length cDNA was sequenced and cloned into the pGEX vector and the recombinant protein expressed in BL21 (DE3) cells and purified. A vaccination trial was performed consisting of a test group (*n* = 8) immunised with recAgs (a mix of two recombinant antigens, Ssλ15 and the previously described Ssλ20∆B3) and a control group (*n* = 8) immunised with PBS. All analyses were performed with R Statistical Environment with α set at 0.050.

**Results:**

The full-length open reading frame of the 1,821 nt cloned cDNA encodes a 64 kDa polypeptide, the sequence of which had 96 % identity with a hypothetical protein of *S. scabiei*. Ssλ15 was localised by immunostaining of skin sections in the tegument surrounding the mouthparts and the coxa in the legs of mites. Rabbit immunisation with recAgs induced high levels of specific IgG (*P* < 0.010) and increased levels of total IgEs. However, no significant clinical protection against *S. scabiei* challenge was detected. Unexpectedly, the group immunised with the recAgs mix had significantly higher lesion scores (*P* = 0.050) although lower mean mite densities than those observed in the control group. These results might indicate that the lesions in the recAgs group were due not only to the mites density but also to an exacerbated immunological response after challenge, which is in agreement with the specific high levels of pro-inflammatory cytokines (IL-1 and TNFα) detected after challenge in this group.

**Conclusions:**

The selected antigens delivered as recombinant proteins had no clinical protective efficacy against *S. scabiei* infestation although immunisation reduced mite density. However, these results pave the way for future studies on alternative production systems, adjuvants, delivery methods and combinations of antigens in order to manage stimulation of clinical protective immune responses.

**Electronic supplementary material:**

The online version of this article (doi:10.1186/s13071-016-1717-9) contains supplementary material, which is available to authorized users.

## Background

Sarcoptic mange (De Geer) (scabies) is a highly contagious parasitic infestation of global distribution, caused by the burrowing mite *S. scabiei* that infests humans and a wide range of domestic and wild mammals [1–3], causing important economic losses.

Acaricides are used to control sarcoptic mange [4–6] but they are expensive and can be highly toxic to the environment, foodstuff and animal handlers. Furthermore, systematic use of acaricides causes development of strong acaricide resistance in scabies mites in humans [7], rabbits [8] and dogs [9]. In this sense, and given that previous studies have shown some degree of protective immune responses developed after *S. scabiei* infestation [10–15], vaccination seems to be a good ecological alternative to the use of acaricides for parasite control. The necessity of an effective vaccine to control and treat this skin disease has been mentioned previously for humans (reviewed in [16]) and animal species [17]. However, effective anti-parasite vaccines against sarcoptic mange have not yet been developed. This is due to multiple factors such as, the complexity of interactions between the parasite and the host’s immune system, the fact that we have yet to understand protective mechanisms employed by the host and the large number of parasite encoded proteins, which makes it very difficult to find proteins that have the capacity to confer protective immunity [17].

The generation of comprehensive expressed sequence tag libraries has enabled the initial characterisation of molecules of interest for diagnostics, vaccines and drug resistance development studies [18]. In this sense, different recombinant antigens have been identified and assayed as vaccine candidates which have not conferred complete protection. Vaccination with a mixture of two fused recombinant antigen portions [Ssag1 (homologous to the house dust mite *Euroglyphus maynei* allergen M-177, an apolipoprotein from hemolymph) and Ssag2] in a rabbit/*S. scabiei* var. *canis* model did not produce reduction in the numbers of mites although rabbits did not exhibit the typical crust characteristics [19]. *Sarcoptes scabiei* glutathione S-transferase, a target for vaccine development in several parasitic diseases, did not induce specific antibodies in mange-infested dogs and pigs [20]. Likewise, vaccination of rabbits with tropomyosin allergen of *S. scabiei*, a protein with proven immunogenic properties, did not efficiently control sarcoptic mange although the lesion areas were smaller at the end of the experiment [21].

The purpose of this study was to identify immunodominant antigens in a *S. scabiei* var. *hominis* cDNA library and to evaluate their potential as vaccine candidates in a rabbit/*S. scabiei* var. *cuniculi* model.

## Methods

### Ethical considerations

Experimental procedures were approved by the SERIDA Animal Ethics Committee and authorised by the Regional Consejería de Agroganadería y Recursos Autoctonos del Principado de Asturias, Spain. Experiments were conducted in accordance with the Spanish and European current legal requirements and guidelines regarding experimentation and Animal Welfare.

### Selection and cloning of recombinant *S. scabiei* antigens

The recombinant DNA techniques and bacteria strains used in this study have been previously described [22].

The *S. scabiei* amplified cDNA library Yv4 used in this study was kindly provided by Dr. David J. Kemp (Malaria and Scabies Laboratory, Queensland Institute of Medical Research, Brisbane, Australia) and contained an average insert size of 1.6 kb cDNAs [23] flanked by *Eco*RI and *Xho*I restriction sites in the vector λZAP express (Stratagene, La Jolla, USA). Two cDNA clones Ssλ20 and Ssλ15 were selected by immunoscreening of the Yv4 library using a 1:20 dilution of a serum taken from a naturally infested chamois and a 1:50 dilution of a serum taken from an experimentally infested rabbit, respectively, following a previously described procedure [24]. The immunocomplexes were detected using a 1:1,500 dilution of protein G or protein A peroxidase-conjugated (Sigma, St. Louis, MO, USA) and 4-chloro-1-naphthol as chromogenic substrate (Sigma, St. Louis, MO, USA). Positive plaques were re-screened at a lower density until pure plaque populations were obtained. This process was followed by *in vivo* excision of pBK-CMV phagemids from the λZAP express vectors following the manufacturer’s instructions.

Cloning, expression and purification of recombinant *S. scabiei* antigen Ssλ20∆B3, the Ssλ20 derivative used in this study, was previously described in [22]. In order to produce the selected *S. scabiei* Ssλ15 specific antigen as a fusion protein with Glutathione S-transferase (GST), the positive clone was excised as a phagemid (pBK-CMV-Ssλ15) which was double-digested with the restriction endonucleases *Eco*RI and *Xho*I. The restriction fragment corresponding to the *S. scabiei* cDNA, was gel-purified and ligated into pGEX-4 T3 digested with the same enzymes (pGEX-4T3-Ssλ15). Sequence analysis of plasmid DNA (pBK-CMV-Ssλ15 and pGEX-4 T3-Ssλ15) was done using the T7 and T3 promoter primers and internal primers Ss5forward (5′-GAG GAA TCG GAT ATG ATT CG-3′), representing nucleotides 617–636 of the cDNA) and Ss6reverse (5′-GAC ATA TTT AGA CAT ATG GC-3′), representing nucleotides 1,161–1,142 of the cDNA) to complete the entire cDNA sequence. Sequencing reactions were done using BigDye® Terminator v3.1 kit and analysed on an ABI PRISM 3100 Genetic Analyser. The nucleotide and deduced amino acid sequences were analysed with Vector NTI (Invitrogen, Carlsbad, California, USA). For sequence similarity the Blast program at the NCBI web server was used. The prediction of transmembrane helices in the deduced polypeptide was carried out by TMHMM Server v. 2.0 (http://www.cbs.dtu.dk/services/TMHMM). The presence of a signal peptide was investigated using SignalP-4.1 at the Centre of Biological Sequence Analysis [25] (http://www.cbs.dtu.dk/services/SignalP-4.1). The hydropathicity plot was calculated according to Kyte & Doolittle [25] using nine residues as window size.

### Purification of recombinant proteins

The recombinant proteins were produced in transformed BL21 *E. coli* cultures induced with 100 μM isopropyl-β-D thiogalactopyranoside (IPTG) for 4 h and purified by affinity chromatography using a Glutathione-Sepharose 4B column (Amersham Biosciences, Barcelona, Spain) according to the manufacturer’s instructions. The sarcoptes-derived polypeptide Ssλ20∆B3 was excised from the GST by thrombin cleavage, while the GST-Ssλ15 was either excised from the GST for preparation of specific antisera or directly eluted from the column as a fusion protein with GST with 50 mM Tris-HCl, 10 mM reduced glutathione, pH 8.0 for its use in the vaccination trial. Proteins were analysed by SDS-PAGE and quantified by the Bradford method [26] using bovine serum albumin as the standard.

### Mass spectrometry analysis of protein spots

The gel bands of interest were manually excised from gels. Proteins selected for analysis were in-gel reduced, alkylated and digested with trypsin according to [27]. Briefly, the samples were reduced with 10 mM dithioerytritol in 25 mM ammonium bicarbonate for 30 min at 56 °C and subsequently alkylated with 55 mM iodoacetamide in 25 mM ammonium bicarbonate for 15 min in the dark. Finally, samples were digested with 12.5 ng/μl sequencing grade trypsin (Roche Molecular Biochemicals, Basel, Switzerland) in 25 mM ammonium bicarbonate (pH 8.5) overnight at 37 °C. After digestion, the supernatant was collected and 1 μl was spotted onto a MALDI target plate and allowed to air-dry at room temperature. Then, 0.6 μl of a 3 mg/ml of α-cyano-4-hydroxy-cinnamic acid matrix (Sigma, St. Louis, MO, USA) in 50 % acetonitrile was added to the dried peptide digest spots and allowed to air-dry again at room temperature. MALDI-TOF MS analyses were performed in a 4800 Plus Proteomics Analyzer MALDI-TOF/TOF mass spectrometer (Applied Biosystems, MDS Sciex, Toronto, Canada) at the Genomics and Proteomics Center, Complutense University of Madrid. The MALDI-TOF/TOF operated in positive reflector mode with an accelerating voltage of 20,000 V. All mass spectra were calibrated internally using peptides from the auto digestion of trypsin. For protein identification UniProt-SwissProt Database (date 14th June 2009; 545,388 sequences; 193,948,795 residues) without taxonomy restriction and a home-made data base with the sequence *S. scabiei* Ss15-2-A protein (1 sequence; 566 residues) were searched using MASCOT v 2.3 (www.matrixscience.com) through the Global Protein Server v 3.6 from ABSCIEX. Search parameters were: carbamidomethyl-Cystein as fixed modification and oxidised Methionine as variable modification, peptide mass tolerance 50 ppm and 1 missed trypsin cleavage site allowed. In all proteins identified, the probability scores were greater than the score fixed by mascot as significant with a *P*-value < 0.050.

### Western blotting

After SDS-polyacrylamide (10 %) gel electrophoresis, the proteins were transferred onto Immobilon-P transfer membranes (Millipore, Billerica, MA, USA) using a Mini Protean II (Bio-Rad, Hercules, CA, USA) electro-blotting apparatus at 100 V for 1 h in 25 mM Tris/192 mM glycine buffer, pH 8.3 containing 20 % methanol. After a blocking step specific antigens were revealed with serum from a mange-infested rabbit serum diluted 1:200, a mange-infested chamois diluted 1:100, an anti-GST monoclonal antibody diluted 1:5,000 (SIGMA, Madrid, Spain), and a mix of a rabbit pre-immune serum (1:100) and a serum from a mange-free chamois (1:200) followed by the addition of the appropriate species-specific peroxidase-conjugated secondary antibody. The immunocomplexes were revealed using 4-chloro-1-naftol as substrate.

### Preparation of antisera

Passive elution of the thrombin excised Ssλ15 70 kDa protein band from polyacrylamide gel pieces was done following the protocol of Thermo Scientific and the efficiency of the process checked analysing the eluted protein on a 10 % SDS-PAGE gel. One New Zealand White rabbit was immunised with the purified protein (70 kDa Ssλ15 gel-eluted polypeptide) for antibody production. The immunisation protocol consisted of 5 intramuscular injections at days 1, 14, 28, 42 and 56, using 200 μg of the purified protein emulsified with incomplete Freund’s adjuvant for the first boost and without adjuvant for the other 4 injections. The serum from the bleed on day 63 and the pre-immune serum from the same rabbit were used for immunolocalisation studies.

### Immunolocalisation of the Ssλ15 encoded antigen

Skin samples from a *S. scabiei* infested chamois were fixed in 10 % neutral formalin and embedded in paraffin using standard procedures. Rabbit skin samples were not used to avoid background reactions as a consequence of using the rabbit antisera for detection. For the immunohistochemical study, 4 μm sections were immunostained using the peroxidase-antiperoxidase (PAP) method [28]. Briefly, the sections were cut, deparaffinised, rehydrated and rinsed with tap water. Afterwards, samples were treated to inactivate the endogenous peroxidase by incubation with methanol containing 3 % H_2_O_2_ for 10 min, washed with water for 10 min and then treated to prevent unspecific binding with a 15 min incubation with 10 % normal swine serum (DAKO, Glostrup, Denmark), 3 % BSA in TBS (5 mM Tris/HCl pH 7.6, 136 mM NaCl). The tissue sections were incubated overnight at 4 °C with a rabbit polyclonal antiserum to the 70 kDa Ssλ15 derived polypeptide diluted 1:700 in TBS and then washed three times with TBS. Then, samples were incubated with swine anti-rabbit serum (DAKO) diluted 1:50 in TBS for 30 min at room temperature and washed three times with TBS followed by incubation with a rabbit PAP (soluble complexes of rabbit antibody to horseradish peroxidase-antihorseradish peroxidase) diluted 1:320 in TBS for 30 min at room temperature. Finally, the sections were incubated with the substrate 3,3′-diaminobenzidine tetrahydrochloride (DAB, Sigma, St. Louis, MO, USA) for 10 min and washed with TBS and water. After staining for 45 s with haematoxylin the preparations were dehydrated, pasted with DPX mountant for histology (Fluka, Sigma, St. Louis, MO, USA) and observed using a light microscope Olympus BH-2 and photographed using a digital camera Olympus DP-12. Pre-immune antisera of the rabbits used to produce the antisera were used as negative controls.

### Vaccination trial and mite challenge

Sixteen, 3 month old scabies-free New Zealand White female rabbits of 2.6–3 kg were housed individually and kept under observation during an acclimatisation period of two weeks. Animals were randomly allocated into two groups (8 rabbits per group): group 1 (recAgs group) was immunised with a mix of Ssλ20∆B3 and GST-Ssλ15 *S. scabiei* recombinant antigens (referred to as recAgs mix) and Quil A adjuvant (Accurate Chemical and Scientific corp., Westbury, USA), and group 2 (control group) with PBS pH 7.5 and Quil A. Each animal was injected subcutaneously in two sites in the back (0.5 ml per site), group 1 with 200 μg of recombinant proteins (100 μg of each) and 100 μg of Quil A, and group 2 with PBS and Quil A (100 μg). The immunisation schedule consisted of four immunisations, at one-week intervals for the first three vaccinations and at three-week interval for the last injection. One week after the last immunisation, all animals were challenged with crusts harbouring approximately 2,500 mixed life-cycle stage mites (3 g) taken from previously infested rabbits, and infestations were allowed to progress for 7 weeks. Mites were inoculated by means of a dressing on the left shaved hind limb (foot area) for 48 h [15].

The *S. scabiei* strain used in this experiment derived from clinically affected wild European rabbits [29] and was maintained in New Zealand White rabbits as the source of mites for the challenge of immunised animals. The inoculum for the challenge was obtained from three affected donor rabbits, which were euthanatised on the day of mite collection. Areas of affected skin were cut into small pieces for the inoculum. For the estimation of the number of mites ten pieces of lesioned skin were weighed, placed in Petri dishes on parafilm and incubated for 24 h at 37 °C to encourage mites to migrate out of the crust of skin. After incubation, the number of mites per gram was counted under a stereoscope and the mean value used as an estimation.

### Antibody levels

In order to assess the humoral immune response (IgG and IgE levels) blood samples were collected from the marginal ear vein prior to vaccination, 6 days after the second dose, 13 days after the third dose, 8 days after the fourth dose (just before challenge) and once per week after infestation. Serum samples were obtained from blood and stored at -20 °C until use.

Circulating rabbit serum antibody levels were analysed with an in house recombinant enzyme-linked immunosorbent assay (ELISA) based on the recombinant antigen Ssλ20∆B3 [30]. Negative and positive controls were included in all plates for normalisation and consisted of a pre-immune serum and a serum collected from a mangy rabbit, respectively. The raw data of the measured OD were normalised as recommended by [31] expressing them as a percentage of the positive control in a ratio correcting for the measured OD of the negative control according to the formula: Relative OD = OD_sample_serum_ - OD_negative_control_/OD_positive_control_ - OD _negative_control_.

The cut-off value (0.03 relative OD) was defined as the mean of the relative OD_450nm_ from 21 scabies-free animals plus three times the standard deviation [32, 33].

Due to the lack of an effective secondary anti-rabbit IgE antibody total levels of IgEs were measured. For the quantitative determination of total rabbit IgE concentrations in serum we used the Rabbit immunoglobulin E (IgE) ELISA Kit (CUSABIO BIOTECH co. distributed by bioNova científica, S.L., Madrid, Spain). Due to economic reasons the analysis was carried out only at four selected time points (before vaccination, prior to challenge, and 1 and 7 weeks post-challenge).

### Cytokine levels

Commercial ELISAs were performed to evaluate serum titres of three cytokines (IL-1, IL-6 and TNF-α) before vaccination, prior to challenge and at weeks 1, 2, 3 and 6 post-challenge. The cytokine ELISAs were performed according to the manufacturer’s instructions (ELISA kit, Uscn Life Science Inc., distributed by bioNova científica, S.L., Madrid, Spain).

### Lesion score

The foot area was chosen for mite inoculation in the challenge as mange lesions in naturally infested rabbits have most frequently been initially observed in the limbs. Afterwards skin lesions caused by mite challenge were assessed for their extension at weekly intervals from weeks 1 to 7 post-infestation. The lesion areas were photographed and measured using a flexible ruler. Lesions were graded as follows: score 0 was assigned if no limb lesions were observed, score 1 when lesions were first observed on the limbs (lesions ≤ 7.75 cm^2^), score 2 when lesions were between 7.75–15.5 cm^2^, score 3 when lesions ranged from 15.5–31 cm^2^ and score 4 when lesions were > 31 cm^2^, following established procedures previously published [15]. The effect of infestation on body condition was also assessed from the changes in the body weight, which was recorded once per week from the beginning of the experiment.

### Mite burden

Seven weeks after challenge rabbits were euthanised by intravenous injection of 0.3 ml per kg of body weight of T-61 Euthanasia Solution® (Intervet) (Embutramide 200 mg; Mebezonium Iodide 50 mg; Tetracaine Hydrochloride 5 mg/ml). Afterwards a fragment of skin was taken from the lesioned area of the left hind limb and stored at -20 °C. For mite counts we followed the protocol described by [34], which allows approximately 88 % recovery of *S. scabiei* mites. Briefly, 2 cm^2^ of skin were cut from the skin fragment, suspended in 4 ml of 10 % KOH solution containing 1 % Tween 80, incubated for 18 ± 2 h at 45 °C, the material was agitated for 2–3 min with a vortex and centrifuged at 500 g for 15 min. Then, the supernatant was decanted to 1 cm above the pellet (about 2.5 ml were removed), which was suspended in the residual liquid, then 2 ml 70 % ethanol were added to rinse the walls of the tube to get a final volume of 4 ml. Twenty replicates of 25 μl digested suspension (a total of 500 μl) per rabbit were observed under the stereomicroscope, the number of mites counted and the total number of mites in the 4 ml was estimated.

### Statistical analysis

All analyses were performed with R Statistical Environment [35], with confidence intervals stated at 95 % (α = 0.050). Differences in mite density were established by Kruskal-Wallis Rank Sum test, as density did not follow normal distribution. Analyses of variance for repeated measures for each dependent variable (IgG levels, IgE levels, cytokines levels, lesion scores and weight) were performed by means of the *ez* package [36]. Data were analysed, using immunisation group and time as fixed factors and the rabbit as a random factor to account for repeated measures variability.

## Results

### Identification and sequence analysis of *S. scabiei* immunodominant antigens

In order to identify *S. scabiei* antigens to use individually or as a mixture as candidates for vaccine development 2 × 10^6^ PFU from the *S. scabiei* var. *hominis* amplified library Yv4 were screened. Two cDNA clones, Ssλ20 and Ssλ15, showing the strongest reactions with sera taken from a naturally infested chamois and an experimentally infested rabbit, respectively, were selected as the most immunodominant.

Selection, cloning, expression, purification and immunolocalisation of recombinant *S. scabiei* antigen Ssλ20∆B3, a truncated derivative of Ssλ20 used in this study, was previously described [22]. The Ssλ20∆B3 cDNA encodes a 28.9 kDa polypeptide of unknown function and contains one out of the 21 amino acid tandem repeats encoded by the original Ssλ20 cDNA clone [22]. Ssλ20∆B3 was detected in Western and dot-blot by sera from an infested chamois [22].

**Fig. 1:**
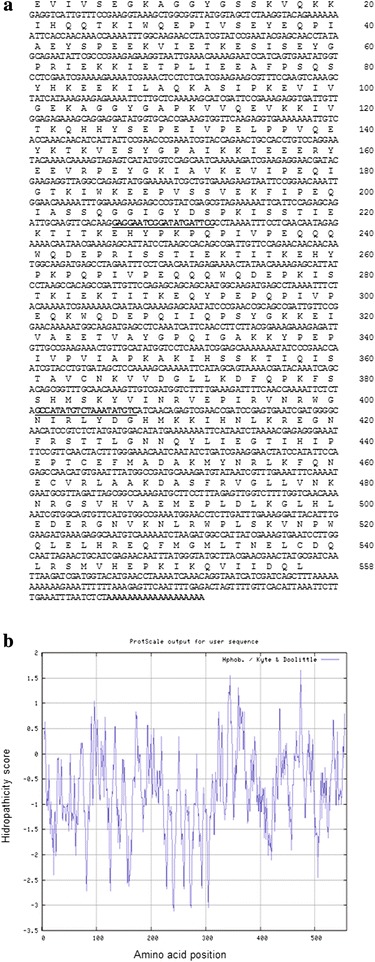
**a** Nucleotide sequence of *S. scabiei* Ssλ15 cDNA (GenBank: KU359774) and amino acid sequence of the predicted translation product. The numbers on the right vertical axis refer to the amino acid position in the Ssλ15 cDNA. The poly (A) tail is shown in boldface. Internal primers Ss5forward (nucleotides 617–636 of the cDNA) and Ss6reverse (nucleotides 1161–1142 of the cDNA) used to complete Ssλ15cDNA nucleotide sequence are boldface and underlined. **b** Hydropathicity profile of Ssλ15 polypeptide

The second immunodominant antigen, Ssλ15, is described here for the first time. The nucleotide sequence of Ssλ15 cDNA clone (Fig. 1a) was 1,821 nt long and the sequence was submitted to the GenBank database on 29th December 2015 (GenBank: KU359774).

Sequence analysis revealed that the Ssλ15 cDNA contained an open reading frame (ORF) starting at cDNA 5′-end and ending at a TAA codon located at nt 1,712–1,714. This cDNA has a 3′-untraslated region of 78 nt preceding the poly (A) tail. This ORF had coding capacity for a 558 amino acid polypeptide with a predicted molecular weight of 64 kDa, and pI 7.36. The most abundant amino acids are Glu (12.2 %), Lys (11.1 %) and Ile (9.9 %). The hydropathicity profile of the deduced protein is shown in Fig. 1b. No polyadenylation signal was detected. No signal peptides or transmembrane domains were evident in the polypeptide. No significant matches for the cDNA nucleotide sequence of Ssλ15 were found in the GenBank non-redundant database (BLASTn search 8th April 2016). However, the deduced amino acid sequence of Ssλ15 antigen had a 96 % of identity (BLASTp search 8th April 2016) with the hypothetical protein QR98_0083330 of *S. scabiei* (GenBank: KPM09788.1) [37], whose function has been provisionally noted as a DNA translocase FTSK.

### Production and characterisation of recombinant Ssλ15

**Fig. 2:**
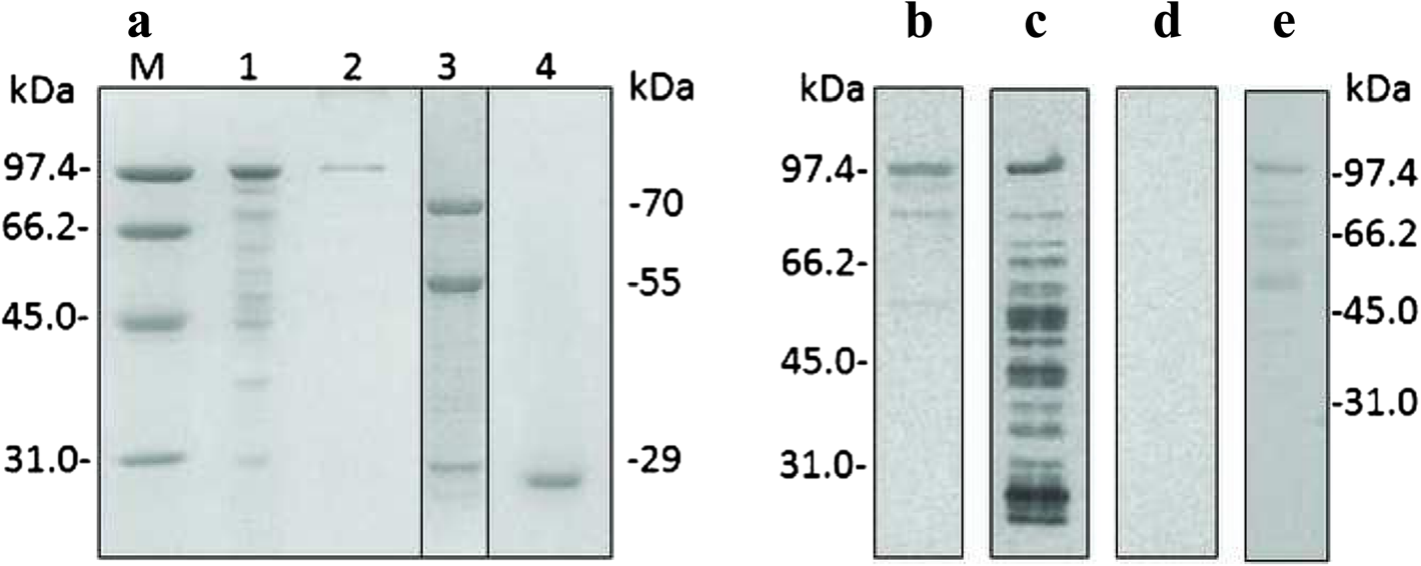
Analysis of Ssλ15 expression and purification. **a** Coomassie Blue stained 10 % SDS-PAGE gel. Lane 1, cell-free extract from pGEX-Ssλ15 transformed *E. coli* BL21 cells loaded to the affinity column; Lane 2, GST- Ssλ15 fusion protein bound to the column; Lane 3, purified Ssλ15 sarcoptes moiety after cleavage with thrombin; Lane 4, GST bound to the column after cleavage; M, low molecular weight protein markers (Amersham Biosciences, Barcelona, Spain). The sample in Lane 3, separated by black lines, was resolved in a different gel from samples M, 1, 2 and 4. **b**-**e** Western blot analysis of GST-Ssλ15 protein using a serum sample from a rabbit experimentally-infested with *S. scabiei* (**b**), an anti-GST monoclonal antibody (**c**), a mix of sera from a non-infested rabbit and a non-infested chamois (**d**) and serum from a mange-infested chamois (**e**). Samples for Western blot analysis shown in **b**, **c** and **d** were resolved on the same gel, in a different gel from sample **e**, which was resolved in an independent gel

In order to facilitate the purification of the selected *S. scabiei* Ssλ15 specific antigen, it was produced as a fusion protein with GST, by inserting the *Eco*RI and *Xho*I digested cDNA into pGEX-4 T3 expression vector. The GST-Ssλ15 fusion protein was expressed in transformed BL21 *E. coli* cultures and purified by affinity chromatography from a cell-free extract (Fig. 2a, Lane 1). SDS-PAGE analysis showed that the molecular weight of the expressed protein was about 98 kDa (Fig. 2a, Lane 2), differing slightly from the estimated theoretical weight of 90 kDa consisting of the predicted 64 kDa Ssλ15 protein and GST (26 kDa). Digestion of the fusion protein with thrombin liberated three proteins of approximately 70 kDa, 60 kDa and 29 kDa from the affinity column (Fig. 2a, Lane 3) while GST stayed bound (Fig. 2a, Lane 4). No other thrombin recognition sites (LVPRGS) apart from the one present in the vector were identified in the polypeptide amino acid sequence. Mass spectrometry analysis of the three protein spots (70, 60 and 29 kDa) revealed that the 60 and 29 kDa bands were fragments of the Ssλ15 70 kDa moiety (see Additional file 1).

The purified fusion protein GST-Ssλ15 was detected in Western blot by antiserum from an experimentally infested rabbit (Fig. 2b), a commercial monoclonal antibody against GST (Fig. 2c), and a naturally infested chamois (Fig. 2e), while no reaction was observed when using a mix of sera of a non-infested rabbit and a non-infested chamois (Fig. 2d). This result confirmed not only the nature of the fusion protein but also that Ssλ15 was the target of an immune response in mange-infested animals.

**Fig. 3:**
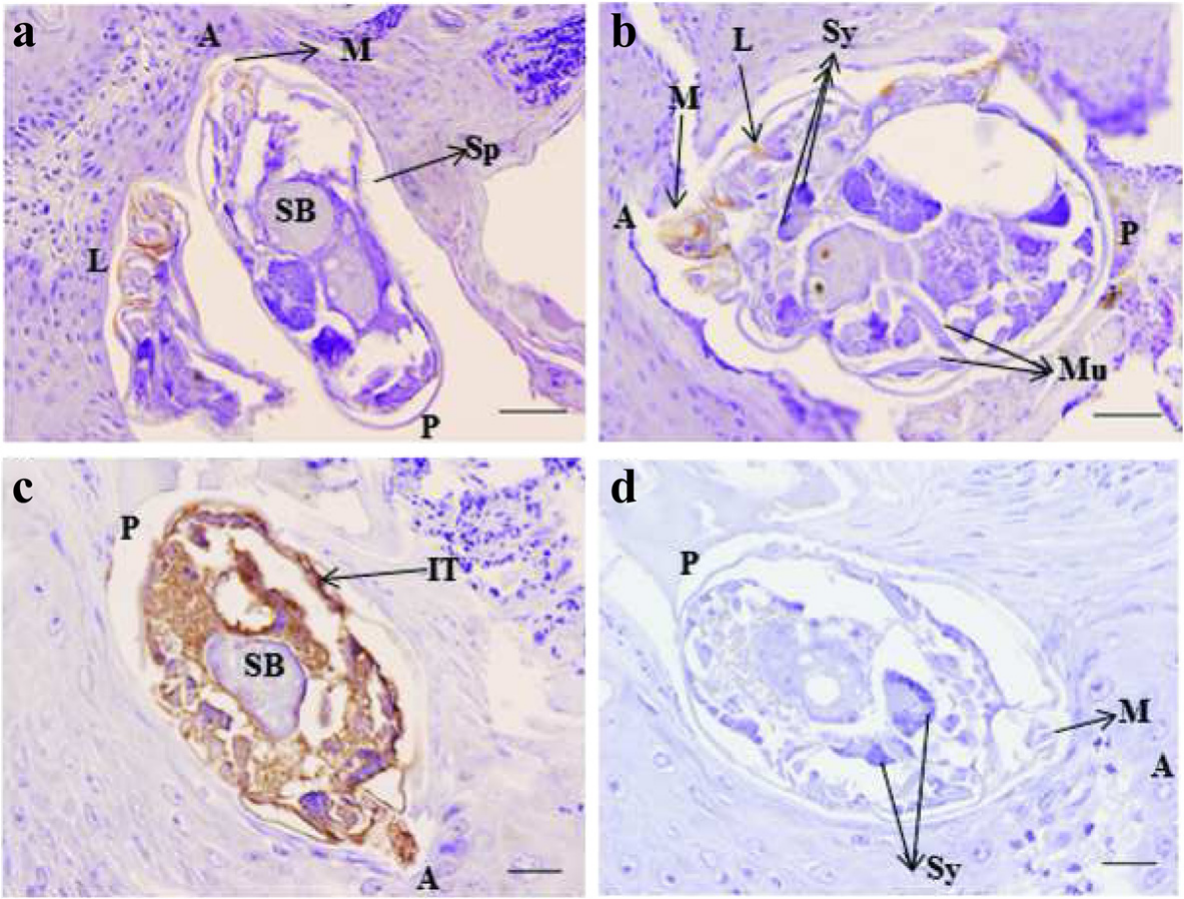
Immunolocalisation of *S. scabiei* Ssλ20∆B3 and Ssλ15 antigens in skin sections derived from a mange-infested chamois. **a**, **b** Peroxidase anti-peroxidase immunostaining after reaction with a rabbit polyclonal anti-Ssλ15 serum, **c** a rabbit polyclonal anti-Ssλ20∆B3 serum, **d** a rabbit pre-immune serum. Ssλ15 immunolabeling is located in the tegument around the mouthparts and around the coxa of the legs (**a** and **b**), and Ssλ20∆B3 was located in the integument and the spaces surrounding the parasite’s vital organs (**c**). No staining is observed in mites and host tissues when using pre-immune sera, confirming that the detected immunolabeling is specific (**d**). *Abbreviations*: A, anterior end of mite; P, posterior end of mite; Sp, spicules; M, mouthparts; L, legs; SB, stomach blocks; IT, the integument of the epidermis; Sy, synganglion; Mu, striated muscle. *Scale-bars*: a, b, 50 μm; c, d, 20 μm

The gel-excised, passive eluted and purified 70 kDa polypeptide was used for the preparation of a specific rabbit antiserum for further polypeptide immunolocalisation in the mite’s body. As shown in Fig. 3a and b, Ssλ15 immunolabeling was located in the tegument around the mouthparts and around the coxa of the legs. For the Ssλ20∆B3 antigen, which forms part of the recAgs vaccine preparation, specific immunostaining is also shown in Fig. 3c. Ssλ20∆B3 was located in the integument and the spaces surrounding the parasite’s vital organs [22]. No staining was observed in host tissues, or when using pre-immune sera, confirming that the detected immunolabeling was specific (Fig. 3d).

### Vaccination trials

In order to determine whether a protective immune response could be elicited using the new recAgs mix a vaccination trial was performed. As described, two groups of eight rabbits were immunised four times with either the recAgs mix or PBS with Quil A adjuvant at one week intervals for the first three immunisations and at three week interval for the last immunisation. One week after the last immunisation, all animals were challenged with crusts containing 2,500 mites taken from previously infested rabbits.

### Antibody responses

**Fig. 4:**
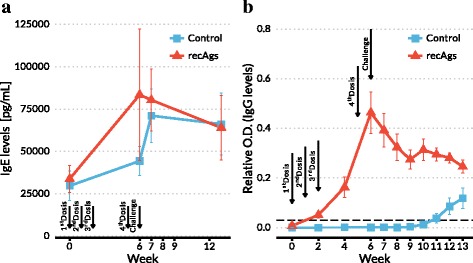
Variation of circulating serum *S. scabiei*-specific IgE (**a**) and total IgG (**b**) antibody levels in sera of immunised rabbits detected by ELISA (Casais et al., 2015). Rabbits were immunised four times (1st, 2nd, 3rd and 4th doses); control refers to the group vaccinated with PBS and Quil A adjuvant and recAgs refers to the group immunised with a mixture of two *S. scabiei* recombinant antigens (Ssλ20∆B3 and GST-Ssλ15) and Quil A. The dashed line represents the cut-off level of the ELISA used to determine the IgG levels (0.03 relative OD_450 nm_). Data points correspond to the mean values, and the error bars represent the standard error. The IgG levels in the recAgs immunised group were significantly higher than in the control (*F*_(1,14)_ = 52.31, *P* < 0.0001), while IgE levels were not significantly different between both groups (*F*_(1,10)_ = 0.406, *P* = 0.538)

Circulating rabbit serum antibody levels (specific IgG and total IgE levels) were analysed by ELISA. Vaccination induced a significant increase in the levels of total IgE, these were quantified before vaccination, prior to challenge, and 1 and 7 weeks post-challenge in the control and vaccinated groups (Fig. 4a). Immunisation of rabbits induced an increase in total IgE levels, which was stronger in the recAgs group. However, *S. scabei* challenge induced a rapid and strong increase in the IgE levels in the control group which was not observed in the recAgs immunised group. One week after challenge a slight decrease of the IgE antibody levels was observed in both groups. No statistically significant differences were observed between either groups (*F*_(1,19)_ = 0.406, *P* = 0.538). Immunisation resulted in specific IgG antibody responses in all animals immunised with the recAgs mix, although levels varied between individuals (Fig. 4b). In the control group, where rabbits received PBS and adjuvant only, IgG levels remain at pre-immunisation levels until challenge when they develop a specific IgG response. In the recAgs group there was a strong IgG response after immunisation, which peaked at week 6 and decreased immediately after challenge with a slight increased at week 4 post-challenge observed. The IgG levels in the recAgs immunised group were significantly higher (*F*_(1,14)_ = 52.31, *P* < 0.0001) than in the control group.

### Cytokines in sera

**Fig. 5:**
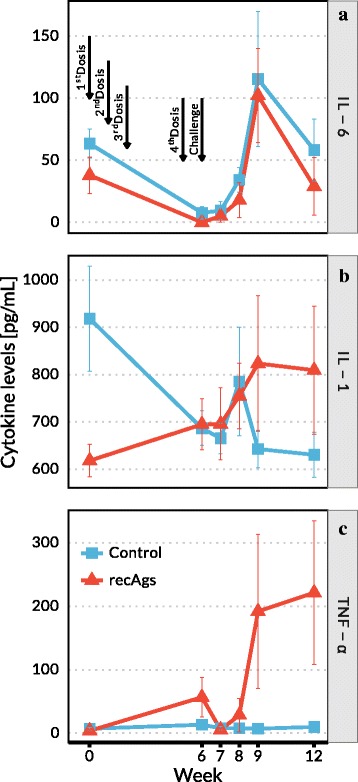
Serum values of IL-6 (**a**), IL-1 (**b**) and TNF-α (**c**) in rabbits immunised and challenged with *S. scabiei.* The kinetics of the cytokines are depicted in separate graphics, where the data points correspond to the mean values of the group at each time point, and the error bars represent the standard error. Rabbits were immunised four times (1st, 2nd, 3rd and 4th doses), control refers to the group vaccinated with PBS and Quil A adjuvant and recAgs refers to the group immunised with a mixture of two *S. scabiei* recombinant antigens (Ssλ20∆B3 and GST-Ssλ15) and Quil A

Systemic immune responses in control and recAgs immunised groups were measured in serum by means of an anti-inflammatory cytokine related with lesions or tissue damage (IL-6) and two pro-inflammatory cytokines (TNF-α, IL-1) before immunisation, prior to challenge and 1, 2, 3 and 6 weeks post-challenge (Fig. 5). The serum kinetics of IL-6 response (Fig. 5a) was similar in both groups, a slight decrease in IL-6 levels was observed after immunisation and a strong increase after challenge which peaked at week 9 (corresponding to week 3 post-challenge). Regarding IL-1 levels (Fig. 5b), rabbits immunised with the recAgs mix exhibited lower levels of this cytokine in serum during immunisation than their control counterparts, however after challenge IL-1 levels were very similar in both experimental groups remaining high in the recAgs immunised group as compared with rabbits in the control group. Finally, the TNF-α profile (Fig. 5c) was rather different, TNF-α levels exhibited a modest increase during the immunisation period (at week 6 TNF-α levels were about 4 times higher in rabbits belonging to the recAgs group as compared with their control counterpart). After challenge a rapid and strong increase in the TNF-α levels was observed in the recAgs group, which from week 3 post-challenge TNF-α levels were around 23 times higher than the control group. No significant differences were observed between groups in the levels of the three cytokines investigated, however in the case of the IL-1 and TNF-α the recAgs immunisation mix used affected significantly the kinetics observed, as the way the levels of these two cytokines evolve through the time in each group is significantly different from the way they evolve in the control group (*F*_(5,60)_ = 3.434, *P* = 0.00854 and *F*_(5,60)_ = 2.662, *P* = 0.0307, respectively).

### Clinical score and mite density

**Fig. 6:**
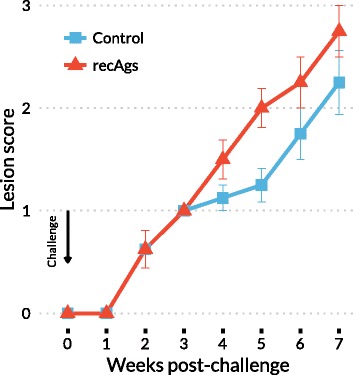
Lesion scores of New Zealand White rabbits immunised and challenged with *S. scabiei.* recAgs refers to the group immunised with a mixture of two *S. scabiei* recombinant antigens (Ssλ20∆B3 and GST-Ssλ15) and Quil A, and Control refers to the group vaccinated with PBS and Quil A adjuvant. Data points correspond to the mean values, and the error bars represent the standard error. The lesions were graded as follows: score 0 was assigned if no limb lesions were observed, score 1 when lesions were first observed on the limbs (lesions ≤ 7.75 cm^2^), score 2 when lesions were between 7.75–15.5 cm^2^, score 3 when lesions ranged from 15.5–31 cm^2^ and score of 4 when lesions were > 31 cm^2^. The group immunised with the recAgs mix had significantly higher lesion scores than the control group (*F*_(1,14)_ = 4.608, *P* = 0.0498) from week 4 to week 6 post-challenge

Finally, the protective effect of the potential vaccine was assessed in the rabbit-*S. scabiei* var. *cuniculi* model by measuring the infested areas over the course of infestation (Fig. 6). All rabbits in the vaccinated and control group developed mange lesions from week 2–3 post-challenge, which progressed slowly and were mainly observed at the site of inoculation and around the nails as rough alopecic areas and parakeratotic crusts. Lesions did not spread to other regions of the body. At seven weeks post-challenge, the severity of lesions varied between animals with the majority of rabbits having lesion scores above 2. Unexpectedly, the group vaccinated with the recAgs mix had significantly higher lesion scores than the control group (*F*_(1,14)_ = 4.608, *P* = 0.0498), as seen in Fig. 6, from week 4 to week 6 post-challenge.

**Fig. 7:**
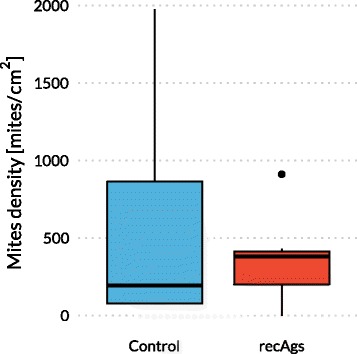
Box-and-whisker plot of mite densities. Control refers to the group vaccinated with PBS and Quil A adjuvant and recAgs refers to the group immunised with a mixture of two *S. scabiei* recombinant antigens (Ssλ20∆B3 and GST-Ssλ15) and Quil A. Box indicates lower and upper quartiles and horizontal line denotes the median of mite density; whiskers indicate the lower and upper extremes; the dot represents an outlier detected in the recAgs group

The density of mites in the skin of all rabbits at week 7 post-challenge was also analysed as an indicator of the protective value of the potential vaccine (Fig. 7). The mean number of mites per cm^2^ was considerably higher in rabbits belonging to the control group (570.06 ± 704.50, ranging from 144 to 3,952 mites) than in rabbits belonging to the recAgs vaccinated group (357.00 ± 272.90, ranging from 224 to 864 mites/cm^2^) with a large variability between individuals within each group. The mean mite densities in the vaccinated group were lower than those observed in the control group, however no significant differences were found between groups (*χ*^2^ = 0.044, *df* = 1, *P* = 0.8335).

No significant effect (*F*_(1,14)_ = 0.091, *P* = 0.768) of the challenge on the weight of the rabbits was observed in either group (data not shown).

## Discussion

In this study, we describe the molecular characterisation of *S. scabiei* Ssλ15 cDNA and the immunolocalisation of the protein encoded by this cDNA in mite infested skin sections. In addition, the protective value of the immune responses developed after immunisation with a new mix of GST-Ssλ15 and the previously described Ssλ20B3 antigen was evaluated in a rabbit/*S. scabiei* var. *cuniculi* model.

Two expressed sequence tags from *S. scabiei,* Ssλ20B3 and Ssλ15, have now been identified by immunoscreening of a *S. scabiei* var. *hominis* library with sera from mangy animals. The two antigens were selected as candidates to include in a potential vaccine because of their strong reaction with sera from infested animals. Moreover, several clones selected during screenings were identified as sequences related to Ssλ15 and Ssλ20, suggesting that these cDNAs encoded immunodominant antigens in infested animals that are derived from abundant parasite mRNAs. The *E. coli* expressed fusion protein GST-Ssλ20B3 was specifically recognised in Western blot with sera of an infested chamois [22] and the GST-Ssλ15 with sera of an infested rabbit and an infested chamois (Fig. 2), which confirms the nature of the fusion proteins and indicates that both polypeptides are the target of immune responses in mange-infested animals.

Attempts to elucidate the function and possible cross-reactivity of Ssλ20 and Ssλ15 with antigens from other related parasites through database searches were performed. They did not show homology to any house dust mite antigens or allergens such as *Dermatophagoides farina*, *D. pteronyssinus* and *E. maynei*. Ssλ15 antigen had a 96 % of identity with a hypothetical protein of *S. scabiei* (GenBank: KPM09788.1) [37], whose function has been provisionally noted as a DNA translocase FTSK. In addition, no positive reactions of Ssλ20B3 with sera from pigs immunised with *D. pteronyssinus* and *Acarus siro* or with sera from tick-infested red deer were detected by ELISA indicating the absence of cross-reactions between Ssλ20B3 and antigens of the mentioned parasites [22, 38]. Cross-reactivity of Ssλ15 with those sera has not been checked because this antigen is not a good candidate to coat ELISA plates for the diagnosis of sarcoptic mange.

Vaccination is the most desirable prophylactic method for any infectious disease. One of the vaccination strategies explored for complex parasites is the use of proteins isolated directly from them. This strategy is advantageous over recombinant proteins in that all structural and immunogenic characteristics that are native to the organism are displayed in the vaccine. However, the availability of native proteins at the required purity and quantity has been the main limiting factor and therefore, production of recombinant antigens has been one of the most common choices to test the protective potential of immunodominant antigens, as was implemented in this study with recAgs (Ssλ20B3 and GST-Ssλ15 mix) in the rabbit/ *S. scabiei* var. *cuniculi* model.

In our system, Ssλ15 antigen was included in the immunisation mixture as a fusion protein with GST to prevent problems derived from thrombin digestion. In addition, GST has immunomodulatory functions and seems a promising vaccine candidate in human schistosomiasis and other parasite infections including scabies [39] so the idea was that the presence of GST could enhance the potential protective immune response elicit by the vaccine preparation.

The experimental design used in this study included two groups, one vaccinated with the recAgs mixture (Ssλ15, Ssλ20ΔB3) plus Quil A adjuvant, and a control group with PBS and Quil A as has been performed in other studies [17, 40]. Nonetheless, this design could have been improved by including a second control group inoculated only with PBS [21]. However, it is possible to conclude that significant differences observed between the recAgs group and the control group are due to the recombinant antigens present in the recAgs preparation and not included in the control group. On the other hand, variations in production of the total IgE and cytokines (IL-1 and IL-6) are observed upon vaccination with adjuvant only so it should be take into account that the effects of the vaccination observed could be due not only to the recombinant antigens but also to Quil A, highlighting the importance of the chosen adjuvant.

Our results show that both defined antigens (Ssλ20B3 and GST-Ssλ15) delivered as recombinant proteins produced in *E. coli* under our vaccination regime of immunisation of rabbits elicited high specific IgGs levels (significantly higher than those observed in the control group) and increased levels of total IgEs (Fig. 4), however in spite of these humoral immune responses, no significant clinical protection against *S. scabiei* challenge was detected. In this sense, the immunological response managed to reduce the number of mites per cm^2^ of skin in the recAgs rabbits but the lesion area score in this group was significantly higher than in the control group, suggesting that mite population might be more sensitive as an indicator of protection than severity of lesions in the vaccination challenge experiment [17]. These results might indicate that the lesions observed in rabbits in the recAgs group were due not only to the mites themselves but also to an exacerbated immunological response after challenge, which is in agreement with the high levels of pro-inflammatory cytokines (IL-1 and TNFα) detected. Examples of a detrimental role of exacerbated pro-inflammatory cytokines have been reported in the literature, particularly in the case of the so called “cytokine storm” after influenza infection or even in vaccination studies in pigs [41, 42].

Both vaccination strategies, the production of recombinant antigens and the use of proteins isolated directly from the parasite, had previously been explored for *S. scabiei* with relative success. Thus, recombinant antigens such as Ssag1 and Ssag2 [19], *S. scabiei* glutathione S-transferase [20] and tropomyosin [21] had been assayed as vaccines and did not confer complete protection against *S. scabiei* challenge. Likewise, while vaccination of goats with soluble or insoluble mite proteins did not produce protective immunity [40], vaccination with a fresh extract from *S. scabiei* conferred partial protection of goats [17], slightly reducing the mite population (*P* = 0.015) but not affecting the severity of lesions, as we have reported in this study in the rabbit model. It has been proposed that the failure to produce complete protection against sarcoptic mange after sensitisation or vaccination may be due to denaturation or degradation of protective antigens, as well as the low abundance or low “immunoprotection” of some of them [17], suggesting that identification and production of proteins having vital function for the mite survival accessible by the host immune system, which constitutes the most important step in vaccine development, will be difficult.

It has been also suggested that the lack of immune protection in goats vaccinated with an extract of *S. scabiei* soluble proteins could be attributed to the absence of protective levels of IgE, indicating that IgE antibody play an important role in immunity to *S. scabiei* infestation [41]. In this regard, our results showed that immunisation with the selected antigen preparation (recAgs) was able to induce high levels of IgG and increased levels of total IgE, with a reduction in the mite population after challenge observed. However, immunisation was not able to reduce lesion areas, which might indicate failure of Ssλ20B3 and GST-Ssλ15 recombinant antigens to elicit protective levels of specific IgE antibody and/or an effective cell mediated response.

The failure to induce complete immune protection in the vaccinated rabbits might indicate that the selected antigens lost their potential native structural and immunogenic properties during the production and purification processes. Immunogenicity is of crucial importance when evaluating expression systems for production of recombinant vaccine antigens [43, 44]. Therefore, we must consider that improvements in expression (for example using virus as expression vectors) and purification strategies may lead to peptides that are structurally more similar to their native counterparts. Likewise, future modifications in our vaccine regime (adjuvants and delivery methods) may direct us towards a more appropriate response. Taking into account the results of our vaccine analysis, the use of this vaccine mix combined with topical creams containing corticoids to control the exacerbated immune response may allow a better control of the disease and reduction in the use of acaricides. It is also possible that the selected antigens are not essential for mite survival and pathogenesis.

## Conclusions

In conclusion, the selected immunodominant antigens (Ssλ15 and Ssλ20∆B3) delivered as recombinant proteins *per se* are not good vaccine candidates against *S. scabiei* infestation in rabbits. Immunisation with a mix of the two antigens induced high levels of humoral responses (IgGs and IgEs), however, immunisation had no clinical protective efficacy (lesion scores were significantly higher although immunisation reduced mite density). Further studies on alternative production systems, adjuvants, delivery methods, immunisation protocols and combinations of antigens will be required to manage stimulation of clinical protective immune responses.

## Abbreviations

ELISA, enzyme-linked immunosorbent assay; IPTG, isopropyl-Beta-D-hiogalactopyranoside; GST, gluthathione s-transferase

## Additional file

Additional file 1: Mass spectrometry results. Results from mass spectrometry analysis of the 70, 60 and 29 kDa protein spots generated by digestion of GST-Ssλ15 with thrombin during the purification process of the Ssλ15 antigen. (PDF 560 kb)
