# Supplementary material for: Vaccination of rabbits with immunodominant antigens from Sarcoptes scabiei induced high levels of humoral responses and pro-inflammatory cytokines but confers limited protection
Source: Parasit Vectors. 2016 Aug 8;9:435. doi: 10.1186/s13071-016-1717-9 (PMC4977775; doi:10.1186/s13071-016-1717-9)
Supplement: Additional file 1: — Mass spectrometry results. Results from mass spectrometry analysis of the 70, 60 and 29 kDa protein spots generated by digestion of GST-Ssλ15 with thrombin during the purification process of the Ssλ15 antigen. (PDF 560 kb) [file 13071_2016_1717_MOESM1_ESM.pdf]

Mascot Search Results for the 70 kDa protein spot.

Protein View

Match to: **P00001** Score: **364** Expect: **4e-037**
Sarcoptes scabiei 15-2-A

Nominal mass (M_r): **64953**; Calculated pI value: **7.36**
NCBI BLAST search of P00001 against nr
Unformatted sequence string for pasting into other applications

Fixed modifications: Carbamidomethyl (C)
Variable modifications: Oxidation (M)
Cleavage by Trypsin: cuts C-term side of KR unless next residue is P
Number of mass values searched: **65**
Number of mass values matched: **37**
Sequence Coverage: **58%**

Matched peptides shown in **Bold Red**

1 **GSPNSARGEV IVSEGKAGGY GSSKVQKKIH QQTK**IWQEPI VSEYEQPIAE
51 YSPEEK**VIET KESISEYGPR IEK**K**IETPLI EEAFPSQSYH KEEK**ILAQK**A**
101 **SIPKEVIVGE KAGGYGAPKV VQEVK**KIVTK QHHYSEPEIV PELPPVQEYK
151 **TKVESYGPAI K**KIEEERYEE VRPEYGKIAV K**EVIPEQIGT KIWKEEPVSS**
201 **VEKFIPEQIA SSQGGIGYDS PK**ISSTIEK**T ITKEHYPKPQ PIVPEQQQWQ**
251 **DEPR**ISSTIE KTITKEHYPK PQPIVPEQQQ WQDEPKISTK IEK**TITKEQY**
301 **PEPQPIVPEQ KWQDEPQIIQ PSYGKKEIVA EETVAYGPQI GAKKYPEPIV**
351 **PVIAPK**AKIH SSKTIQISTA VCNKVVDGLL KDFQPKFSSH MSK**YVINRVE**
401 **PIRVNR**WGNI R**LYDGHMKK**I HNLKREGNFR STTLGNNQYL IEXTIHIPEP
451 TCEFMADAKM YNR**LKFQNEC VRLAAKDASF RVGLLVNKNR GSVHVAEMEP**
501 **LDLKGLHLED ERGNVK**NLRW PLSK**VNPWQL ELHR**EQFMGM LTNELCDQLR

551 SMVHEPKIKQ VIIDQL

Show predicted peptides also

Sort Peptides By ◉ Residue Number ○ Increasing Mass ○ Decreasing Mass

Start - End	Observed	Mr(expt)	Mr(calc)	ppm	Miss	Sequence
1 - 16	1586.8413	1585.8340	1585.8060	18	1	-.GSPNSARGEVIVSEGK.A
8 - 16	917.4628	916.4555	916.4866	-34	0	R.GEVIVSEGK.A
17 - 27	1081.5577	1080.5504	1080.5564	-5	1	K.AGGYGSSKVQK.K
28 - 34	882.4788	881.4715	881.5083	-42	1	K.KIHQQTK.I
57 - 70	1607.8561	1606.8488	1606.8202	18	1	K.VIETKESISEYGPR.I
62 - 70	1037.4857	1036.4784	1036.4825	-4	0	K.ESISEYGPR.I
62 - 73	1407.7349	1406.7276	1406.7041	17	1	K.ESISEYGPRIEK.K
75 - 94	2375.1550	2374.1477	2374.1692	-9	1	K.IETPLIEEAFPSQSYHKEEK.I
100 - 111	1269.7164	1268.7091	1268.7340	-20	1	K.ASIPKEVIVGEK.A
112 - 125	1402.7788	1401.7715	1401.7616	7	1	K.AGGYGAPKVVQEVK.K
151 - 161	1192.6603	1191.6530	1191.6499	3	1	K.TKVESYGPAIK.K
153 - 161	963.5042	962.4969	962.5073	-11	0	K.VESYGPAIK.K
182 - 191	1113.6010	1112.5937	1112.6077	-13	0	K.EVIPEQIGTK.I
192 - 203	1430.7605	1429.7532	1429.7453	6	1	K.IWKEEPVSSVEK.F
204 - 222	1994.0001	1992.9928	1992.9793	7	0	K.FIPEQIASSQGGIGYDSPK.I
230 - 254	3072.4094	3071.4021	3071.5465	-47	1	K.TITKEHYPKPQPIVPEQQQWQDEPR.I
234 - 254	2629.2224	2628.2151	2628.2721	-22	0	K.EHYPKPQPIVPEQQQWQDEPR.I
294 - 311	2125.1133	2124.1060	2124.1103	-2	1	K.TITKEQYPEPQPIVPEQK.W
298 - 311	1681.8635	1680.8562	1680.8359	12	0	K.EQYPEPQPIVPEQK.W
312 - 326	1816.9390	1815.9317	1815.9155	9	1	K.WQDEPQIIQPSYGKK.E
327 - 343	1774.9340	1773.9267	1773.9149	7	0	K.EIVAEETVAYGPQIGAK.K
344 - 356	1450.8812	1449.8739	1449.8595	10	1	K.KYPEPIVPVIAPK.A
394 - 403	1258.7399	1257.7326	1257.7193	11	1	K.YVINRVEPIR.V
399 - 406	982.5609	981.5536	981.5719	-19	1	R.VEPIRVNR.W
412 - 418	863.3719	862.3646	862.4007	-42	0	R.LYDGHMK.K
412 - 419	991.4843	990.4770	990.4957	-19	1	R.LYDGHMKK.I
464 - 472	1193.6431	1192.6358	1192.6023	28	1	R.LKFQNECVR.L
466 - 472	952.4196	951.4123	951.4232	-11	0	K.FQNECVR.L
473 - 481	978.5264	977.5191	977.5294	-11	1	R.LAAKDASFR.V
482 - 490	1012.6123	1011.6050	1011.6189	-14	1	R.VGLLVNKNR.G
489 - 504	1794.9377	1793.9304	1793.9094	12	1	K.NRGSVHVAEMEPLDLK.G
491 - 504	1524.7976	1523.7903	1523.7654	16	0	R.GSVHVAEMEPLDLK.G
491 - 504	1540.7906	1539.7833	1539.7603	15	0	R.GSVHVAEMEPLDLK.G Oxidation (M)
505 - 512	968.4617	967.4544	967.4723	-18	0	K.GLHLEDER.G

505 - 516	1366.7288	1365.7215	1365.7001	16	1	K.GLHLEDERGNVK.N
525 - 534	1291.7079	1290.7006	1290.6833	13	0	K.VNPWQLELHR.E
551 - 559	1068.5754	1067.5681	1067.5797	-11	1	R.SMVHEPKIK.Q

No match to: 855.0230, 861.0355, 877.0079, 892.9871, 906.4780, 908.5552, 950.4529, 966.4337, 1034.1306, 1050.1056, 1052.1069, 1066.0786, 1068.0795, 1153.5757, 1179.6180, 1207.6381, 1307.7120, 1323.7012, 1348.7206, 1535.8325, 1627.9349, 1971.0972, 1978.0656, 1980.0605, 2054.0313, 2163.0508, 2465.2742, 2503.2300

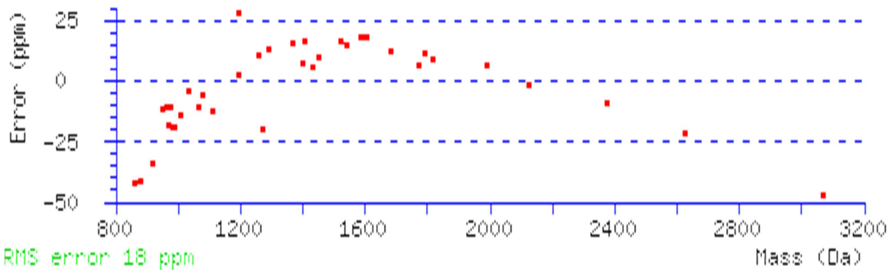

Mascot Search Results for the 70 kDa protein spot

User :
Email :
Search title : SampleSetID: 304, AnalysisID: 3076, MaldiWellID: 36630, SpectrumID: 74703, Path=\CNB_141104\Lola141104 MS\14-120 Rosa su db
Database : Sarcoptes Rosa (1 sequences; 566 residues)
Timestamp : 11 Nov 2014 at 12:13:36 GMT
Top Score : 364 for P00001, Sarcoptes scabiei 15-2-A

Mascot Score Histogram

Protein score is -10*Log(P), where P is the probability that the observed match is a random event. Protein scores greater than 13 are significant ($p<0.05$).

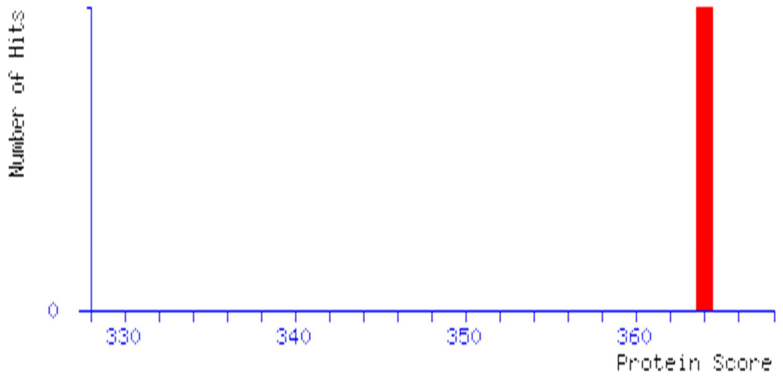

Protein Summary Report

Format As	Protein Summary	Help
	Significance threshold p< 0.05	Max. number of hits 20

Re-Search All

Index

	Accession	Mass	Score	Description
1.	**P00001**	64953	364	Sarcoptes scabiei 15-2-A

Results List

1. P00001 **Mass:** 64953 **Score:** **364** **Expect:** 4e-037 **Matches:** 37

Sarcoptes scabiei 15-2-A

Observed	Mr(expt)	Mr(calc)	ppm	Start		End	Miss	Peptide
863.3719	862.3646	862.4007	-41.84	412	-	418	0	R.LYDGHMK.K
882.4788	881.4715	881.5083	-41.70	28	-	34	1	K.KIHQQTK.I
917.4628	916.4555	916.4866	-33.86	8	-	16	0	R.GEVIVSEGK.A
952.4196	951.4123	951.4232	-11.48	466	-	472	0	K.FQNECVR.L
963.5042	962.4969	962.5073	-10.76	153	-	161	0	K.VESYGPAIK.K
968.4617	967.4544	967.4723	-18.47	505	-	512	0	K.GLHLEDER.G
978.5264	977.5191	977.5294	-10.52	473	-	481	1	R.LAAKDASFR.V
982.5609	981.5536	981.5719	-18.66	399	-	406	1	R.VEPIRVNR.W
991.4843	990.4770	990.4957	-18.82	412	-	419	1	R.LYDGHMKK.I
1012.6123	1011.6050	1011.6189	-13.71	482	-	490	1	R.VGLLVNKNR.G
1037.4857	1036.4784	1036.4825	-3.95	62	-	70	0	K.ESISEYGPR.I
1068.5754	1067.5681	1067.5797	-10.87	551	-	559	1	R.SMVHEPKIK.Q
1081.5577	1080.5504	1080.5564	-5.50	17	-	27	1	K.AGGYGSSKVQK.K
1113.6010	1112.5937	1112.6077	-12.59	182	-	191	0	K.EVIPEQIGTK.I
1192.6603	1191.6530	1191.6499	2.61	151	-	161	1	K.TKVESYGPAIK.K
1193.6431	1192.6358	1192.6023	28.1	464	-	472	1	R.LKFQNECVR.L
1258.7399	1257.7326	1257.7193	10.6	394	-	403	1	K.YVINRVEPIR.V
1269.7164	1268.7091	1268.7340	-19.59	100	-	111	1	K.ASIPKEVIVGEK.A
1291.7079	1290.7006	1290.6833	13.4	525	-	534	0	K.VNPWQLELHR.E
1366.7288	1365.7215	1365.7001	15.7	505	-	516	1	K.GLHLEDERGNVK.N
1402.7788	1401.7715	1401.7616	7.07	112	-	125	1	K.AGGYGAPKVVQEVK.K
1407.7349	1406.7276	1406.7041	16.7	62	-	73	1	K.ESISEYGPRIEK.K
1430.7605	1429.7532	1429.7453	5.56	192	-	203	1	K.IWKEEPVSSVEK.F
1450.8812	1449.8739	1449.8595	9.94	344	-	356	1	K.KYPEPIVPVIAPK.A
1524.7976	1523.7903	1523.7654	16.4	491	-	504	0	R.GSVHVAEMEPLDLK.G
1540.7906	1539.7833	1539.7603	15.0	491	-	504	0	R.GSVHVAEMEPLDLK.G + Oxidation (M)
1586.8413	1585.8340	1585.8060	17.7	1	-	16	1	-.GSPNSARGEVIVSEGK.A
1607.8561	1606.8488	1606.8202	17.8	57	-	70	1	K.VIETKESISEYGPR.I

1681.8635	1680.8562	1680.8359	12.1	298 - 311	0	K.EQYPEPQPIVPEQK.W
1774.9340	1773.9267	1773.9149	6.69	327 - 343	0	K.EIVAEETVAYGPQIGAK.K
1794.9377	1793.9304	1793.9094	11.7	489 - 504	1	K.NRGSVHVAEMEPLDLK.G
1816.9390	1815.9317	1815.9155	8.92	312 - 326	1	K.WQDEPQIIQPSYGKK.E
1994.0001	1992.9928	1992.9793	6.80	204 - 222	0	K.FIPEQIASSQGGIGYDSPK.I
2125.1133	2124.1060	2124.1103	-1.99	294 - 311	1	K.TITKEQYPEPQPIVPEQK.W
2375.1550	2374.1477	2374.1692	-9.05	75 - 94	1	K.IETPLIEEAFPSQSYHKEEK.I
2629.2224	2628.2151	2628.2721	-21.67	234 - 254	0	K.EHYPKPQPIVPEQQQWQDEPR.I
3072.4094	3071.4021	3071.5465	-46.99	230 - 254	1	K.TITKEHYPKPQPIVPEQQQWQDEPR.I

No match to: 855.0230, 861.0355, 877.0079, 892.9871, 906.4780, 908.5552, 950.4529, 966.4337, 1034.1306, 1050.1056, 1052.1069, 1066.0786, 1068.0795, 1153.5757, 1179.6180, 1207.6381, 1307.7120, 1323.7012, 1348.7206, 1535.8325, 1627.9349, 1971.0972, 1978.0656, 1980.0605, 2054.0313, 2163.0508, 2465.2742, 2503.2300

Search Parameters

Type of search : **Peptide Mass Fingerprint**
Enzyme : **Trypsin**
Fixed modifications : **Carbamidomethyl (C)**
Variable modifications : **Oxidation (M)**
Mass values : **Monoisotopic**
Protein Mass : **Unrestricted**
Peptide Mass Tolerance : **± 50 ppm**
Peptide Charge State : **1+**
Max Missed Cleavages : **1**
Number of queries : **65**

Mascot Search Results for the 60 kDa protein spot

Protein View

Match to: **P00001** Score: **236** Expect: **2.5e-024**
Sarcoptes scabiei 15-2-A

Nominal mass (M_r): **64953**; Calculated pI value: **7.36**
NCBI BLAST search of P00001 against nr
Unformatted sequence string for pasting into other applications

Fixed modifications: Carbamidomethyl (C)
Variable modifications: Oxidation (M)
Cleavage by Trypsin: cuts C-term side of KR unless next residue is P
Number of mass values searched: **65**
Number of mass values matched: **26**
Sequence Coverage: **49%**

Matched peptides shown in **Bold Red**

```
  1 GSPNSARGEV IVSEGKAGGY GSSKVQKKIH QQTKIWQEPI VSEYEQPIAE
 51 YSPEEKVIET KESISEYGPR IEKKIETPLI EEAFPSQSYH KEEKILAQKA
101 SIPKEVIVGE KAGGYGAPKV VQEVKKIVTK QHHYSEPEIV PELPPVQEYK
151 TKVESYGPAI KKIEEERYEE VRPEYGKIAV KEVIPEQIGT KIWKEEPVSS
201 VEKFIPEQIA SSQGGIGYDS PKISSTIEKT ITKEHYPKPQ PIVPEQQQWQ
251 DEPRISSTIE KTITKEHYPK PQPIVPEQQQ WQDEPKISTK IEKTITKEQY
301 PEPQPIVPEQ KWQDEPQIIQ PSYGKKEIVA EETVAYGPQI GAKKYPEPIV
351 PVIAPKAKIH SSKTIQISTA VCNKVVDGLL KDFQPKFSSH MSKYVINRVE
401 PIRVNRWGNI RLYDGHMKKI HNLKREGNFR STTLGNNQYL IEXTIHIPEP
451 TCEFMADAKM YNRLKFQNEC VRLAAKDASF RVGLLVNKNR GSVHVAEMEP
501 LDLKGLHLED ERGNVKNLRW PLSKVNPWQL ELHREQFMGM LTNELCDQLR
551 SMVHEPKIKQ VIIDQL
```

Show predicted peptides also

Sort Peptides By ◉ Residue Number ○ Increasing Mass ○ Decreasing Mass

Start - End	Observed	Mr(expt)	Mr(calc)	ppm	Miss	Sequence
1 - 16	1586.8196	1585.8123	1585.8060	4	1	-.GSPNSARGEVIVSEGK.A
8 - 16	917.4770	916.4697	916.4866	-18	0	R.GEVIVSEGK.A
8 - 24	1624.8114	1623.8041	1623.8104	-4	1	R.GEVIVSEGKAGGYGSSK.V
17 - 27	1081.5532	1080.5459	1080.5564	-10	1	K.AGGYGSSKVQK.K
28 - 34	882.4955	881.4882	881.5083	-23	1	K.KIHQQTK.I
35 - 56	2664.2747	2663.2674	2663.2642	1	0	K.IWQEPIVSEYEQPIAEYSPEEK.V
57 - 70	1607.8368	1606.8295	1606.8202	6	1	K.VIETKESISEYGPR.I
62 - 70	1037.4823	1036.4750	1036.4825	-7	0	K.ESISEYGPR.I
62 - 73	1407.7164	1406.7091	1406.7041	4	1	K.ESISEYGPRIEK.K
75 - 94	2375.1843	2374.1770	2374.1692	3	1	K.IETPLIEEAFPSQSYHKEEK.I
100 - 111	1269.7019	1268.6946	1268.7340	-31	1	K.ASIPKEVIVGEK.A
105 - 119	1474.7914	1473.7841	1473.7827	1	1	K.EVIVGEKAGGYGAPK.V
112 - 125	1402.7650	1401.7577	1401.7616	-3	1	K.AGGYGAPKVVQEVK.K
151 - 161	1192.6495	1191.6422	1191.6499	-6	1	K.TKVESYGPAIK.K
153 - 161	963.4922	962.4849	962.5073	-23	0	K.VESYGPAIK.K
163 - 177	1925.9352	1924.9279	1924.9166	6	1	K.IEEERYEEVRPEYGK.I
178 - 191	1524.8541	1523.8468	1523.8923	-30	1	K.IAVKEVIPEQIGTK.I
182 - 191	1113.5953	1112.5880	1112.6077	-18	0	K.EVIPEQIGTK.I
192 - 203	1430.7456	1429.7383	1429.7453	-5	1	K.IWKEEPVSSVEK.F
204 - 222	1993.9891	1992.9818	1992.9793	1	0	K.FIPEQIASSQGGIGYDSPK.I
234 - 254	2629.2996	2628.2923	2628.2721	8	0	K.EHYPKPQPIVPEQQQWQDEPR.I
294 - 311	2125.1187	2124.1114	2124.1103	1	1	K.TITKEQYPEPQPIVPEQK.W
312 - 326	1816.9272	1815.9199	1815.9155	2	1	K.WQDEPQIIQPSYGKK.E
327 - 343	1774.9164	1773.9091	1773.9149	-3	0	K.EIVAEETVAYGPQIGAK.K
344 - 356	1450.8578	1449.8505	1449.8595	-6	1	K.KYPEPIVPVIAPK.A
412 - 418	863.4255	862.4182	862.4007	20	0	R.LYDGHMK.K

No match to: 850.3419, 851.3163, 855.0344, 859.5169, 861.0443, 873.4307, 884.4807, 906.4867, 980.4589, 1012.1168, 1019.4736, 1027.5044, 1034.1140, 1035.1183, 1036.1166, 1050.0912, 1103.5352, 1117.4895, 1135.5591, 1153.5601, 1174.6219, 1190.6023, 1200.6863, 1207.6233, 1209.6469, 1223.1378, 1229.5122, 1252.6464, 1334.6674, 1530.8507, 1535.8148, 1971.0892, 1978.0637, 1980.0552, 2054.0339, 2163.0532, 2185.0513, 2465.3191, 2503.2815

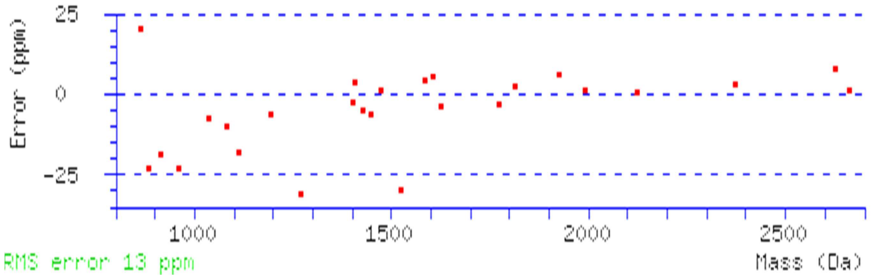

Mascot Search Results for the 60 kDa protein spot

```
User           :
Email          :
Search title   : SampleSetID: 304, AnalysisID: 3076, MaldiWellID: 36631, SpectrumID: 74704,
Path=\CNB_141104\Lola141104 MS\14-120 Rosa   su db
Database       : Sarcoptes Rosa (1 sequences; 566 residues)
Timestamp      : 11 Nov 2014 at 12:13:39 GMT
Top Score      : 236 for P00001, Sarcoptes scabiei 15-2-A
```

Mascot Score Histogram

Protein score is -10*Log(P), where P is the probability that the observed match is a random event.

Protein scores greater than 13 are significant (p<0.05).

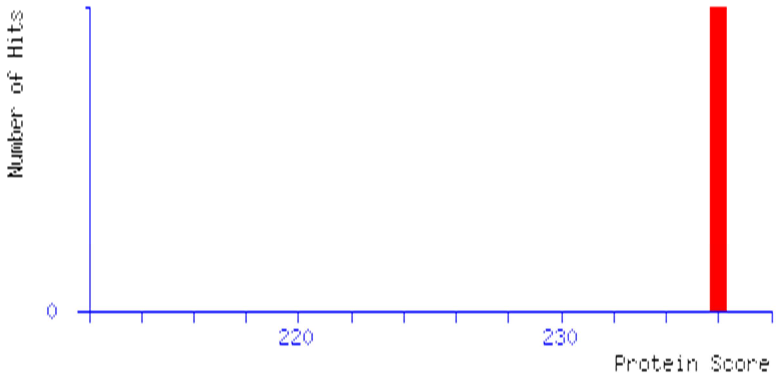

Protein Summary Report

Help

Significance threshold p< 0.05 Max. number of hits 20

Index

	Accession	Mass	Score	Description
1.	P00001	64953	236	Sarcoptes scabiei 15-2-A

Results List

1. P00001 **Mass:** 64953 **Score:** **236** **Expect:** 2.5e-024 **Matches:** 26

Sarcoptes scabiei 15-2-A

Observed	Mr(expt)	Mr(calc)	ppm	Start		End	Miss	Peptide
863.4255	862.4182	862.4007	20.3	412	-	418	0	R.LYDGHMK.K
882.4955	881.4882	881.5083	-22.76	28	-	34	1	K.KIHQQTK.I
917.4770	916.4697	916.4866	-18.37	8	-	16	0	R.GEVIVSEGK.A
963.4922	962.4849	962.5073	-23.22	153	-	161	0	K.VESYGPAIK.K
1037.4823	1036.4750	1036.4825	-7.23	62	-	70	0	K.ESISEYGPR.I
1081.5532	1080.5459	1080.5564	-9.66	17	-	27	1	K.AGGYGSSKVQK.K
1113.5953	1112.5880	1112.6077	-17.71	182	-	191	0	K.EVIPEQIGTK.I
1192.6495	1191.6422	1191.6499	-6.46	151	-	161	1	K.TKVESYGPAIK.K
1269.7019	1268.6946	1268.7340	-31.02	100	-	111	1	K.ASIPKEVIVGEK.A
1402.7650	1401.7577	1401.7616	-2.77	112	-	125	1	K.AGGYGAPKVVQEVK.K
1407.7164	1406.7091	1406.7041	3.56	62	-	73	1	K.ESISEYGPRIEK.K
1430.7456	1429.7383	1429.7453	-4.86	192	-	203	1	K.IWKEEPVSSVEK.F
1450.8578	1449.8505	1449.8595	-6.20	344	-	356	1	K.KYPEPIVPVIAPK.A

1474.7914	1473.7841	1473.7827	0.95	105 - 119	1	K.EVIVGEKAGGYGAPK.V
1524.8541	1523.8468	1523.8923	-29.82	178 - 191	1	K.IAVKEVIPEQIGTK.I
1586.8196	1585.8123	1585.8060	4.00	1 - 16	1	-.GSPNSARGEVIVSEGK.A
1607.8368	1606.8295	1606.8202	5.79	57 - 70	1	K.VIETKESISEYGPR.I
1624.8114	1623.8041	1623.8104	-3.87	8 - 24	1	R.GEVIVSEGKAGGYGSSK.V
1774.9164	1773.9091	1773.9149	-3.23	327 - 343	0	K.EIVAEETVAYGPQIGAK.K
1816.9272	1815.9199	1815.9155	2.42	312 - 326	1	K.WQDEPQIIQPSYGKK.E
1925.9352	1924.9279	1924.9166	5.88	163 - 177	1	K.IEEERYEEVRPEYGK.I
1993.9891	1992.9818	1992.9793	1.29	204 - 222	0	K.FIPEQIASSQGGIGYDSPK.I
2125.1187	2124.1114	2124.1103	0.55	294 - 311	1	K.TITKEQYPEPQPIVPEQK.W
2375.1843	2374.1770	2374.1692	3.29	75 - 94	1	K.IETPLIEEAFPSQSYHKEEK.I
2629.2996	2628.2923	2628.2721	7.70	234 - 254	0	K.EHYPKPQPIVPEQQQWQDEPR.I
2664.2747	2663.2674	2663.2642	1.20	35 - 56	0	K.IWQEPIVSEYEQPIAEYSPEEK.V

No match to: 850.3419, 851.3163, 855.0344, 859.5169, 861.0443, 873.4307, 884.4807, 906.4867, 980.4589, 1012.1168, 1019.4736, 1027.5044, 1034.1140, 1035.1183, 1036.1166, 1050.0912, 1103.5352, 1117.4895, 1135.5591, 1153.5601, 1174.6219, 1190.6023, 1200.6863, 1207.6233, 1209.6469, 1223.1378, 1229.5122, 1252.6464, 1334.6674, 1530.8507, 1535.8148, 1971.0892, 1978.0637, 1980.0552, 2054.0339, 2163.0532, 2185.0513, 2465.3191, 2503.2815

Search Parameters

```
Type of search         : Peptide Mass Fingerprint
Enzyme                 : Trypsin
Fixed modifications    : Carbamidomethyl (C)
Variable modifications : Oxidation (M)
Mass values            : Monoisotopic
Protein Mass           : Unrestricted
Peptide Mass Tolerance : ± 50 ppm
Peptide Charge State   : 1+
Max Missed Cleavages   : 1
Number of queries      : 65
```

Mascot Search Results for the 29 kDa protein spot

Protein View

Match to: **P00001** Score: **67** Expect: **2.1e-007**
Sarcoptes scabiei 15-2-A

Nominal mass (M_r): **64953**; Calculated pI value: **7.36**
NCBI BLAST search of P00001 against nr
Unformatted sequence string for pasting into other applications

Fixed modifications: Carbamidomethyl (C)
Variable modifications: Oxidation (M)
Cleavage by Trypsin: cuts C-term side of KR unless next residue is P
Number of mass values searched: **65**
Number of mass values matched: **15**
Sequence Coverage: **19%**

Matched peptides shown in **Bold Red**

1 **GSPNSARGEV IVSEGK**AGGY GSSKVQKKIH QQTKIWQEPI VSEYEQPIAE

```
 51 YSPEEKVIET KESISEYGPR IEKKIETPLI EEAFPSQSYH KEEKILAQKA
101 SIPKEVIVGE KAGGYGAPKV VQEVKKIVTK QHHYSEPEIV PELPPVQEYK
151 TKVESYGPAI KKIEEERYEE VRPEYGKIAV KEVIPEQIGT KIWKEEPVSS
201 VEKFIPEQIA SSQGGIGYDS PKISSTIEKT ITKEHYPKPQ PIVPEQQQWQ
251 DEPRISSTIE KTITKEHYPK PQPIVPEQQQ WQDEPKISTK IEKTITKEQY
301 PEPQPIVPEQ KWQDEPQIIQ PSYGKKEIVA EETVAYGPQI GAKKYPEPIV
351 PVIAPKAKIH SSKTIQISTA VCNKVVDGLL KDFQPKFSSH MSKYVINRVE
401 PIRVNRWGNI RLYDGHMKKI HNLKREGNFR STTLGNNQYL IEXTIHIPEP
451 TCEFMADAKM YNRLKFQNEC VRLAAKDASF RVGLLVNKNR GSVHVAEMEP
501 LDLKGLHLED ERGNVKNLRW PLSKVNPWQL ELHREQFMGM LTNELCDQLR
551 SMVHEPKIKQ VIIDQL
```

Show predicted peptides also

Sort Peptides By ◉ Residue Number ○ Increasing Mass ○ Decreasing Mass

Start - End	Observed	Mr(expt)	Mr(calc)	ppm	Miss	Sequence	
1 - 16	1586.8153	1585.8080	1585.8060	1	1	-.GSPNSARGEVIVSEGK.A	
57 - 70	1607.8307	1606.8234	1606.8202	2	1	K.VIETKESISEYGPR.I	
62 - 70	1037.4796	1036.4723	1036.4825	-10	0	K.ESISEYGPR.I	
62 - 73	1407.7124	1406.7051	1406.7041	1	1	K.ESISEYGPRIEK.K	
394 - 403	1258.7209	1257.7136	1257.7193	-5	1	K.YVINRVEPIR.V	
412 - 418	863.3875	862.3802	862.4007	-24	0	R.LYDGHMK.K	
464 - 472	1193.6104	1192.6031	1192.6023	1	1	R.LKFQNECVR.L	
466 - 472	952.4219	951.4146	951.4232	-9	0	K.FQNECVR.L	
473 - 481	978.5259	977.5186	977.5294	-11	1	R.LAAKDASFR.V	
491 - 504	1524.7672	1523.7599	1523.7654	-4	0	R.GSVHVAEMEPLDLK.G	
491 - 504	1540.7590	1539.7517	1539.7603	-6	0	R.GSVHVAEMEPLDLK.G	Oxidation (M)
505 - 512	968.4672	967.4599	967.4723	-13	0	K.GLHLEDER.G	
505 - 516	1366.7078	1365.7005	1365.7001	0	1	K.GLHLEDERGNVK.N	
525 - 534	1291.6788	1290.6715	1290.6833	-9	0	K.VNPWQLELHR.E	
551 - 559	1068.5726	1067.5653	1067.5797	-13	1	R.SMVHEPKIK.Q	

No match to: 850.4409, 851.3151, 859.5217, 903.5519, 905.4117, 906.4853, 966.4380, 982.5284, 1000.5289, 1012.1270, 1013.1278, 1014.1285, 1034.1184, 1035.1215, 1036.1210, 1050.0966, 1050.5487, 1077.4968, 1117.5150, 1129.6157, 1153.5623, 1163.5876, 1175.5601, 1182.6327, 1184.6357, 1190.5997, 1207.6219, 1223.1538, 1224.1543, 1225.1528, 1229.5223, 1245.1344, 1246.4282, 1296.6621, 1306.6605, 1320.6833, 1336.6720, 1348.7004, 1364.6843, 1388.6844, 1412.1792, 1414.1847, 1420.6364, 1423.6693, 1465.7380, 1546.7543, 1749.9333, 2163.0525, 2185.0447, 2216.9685

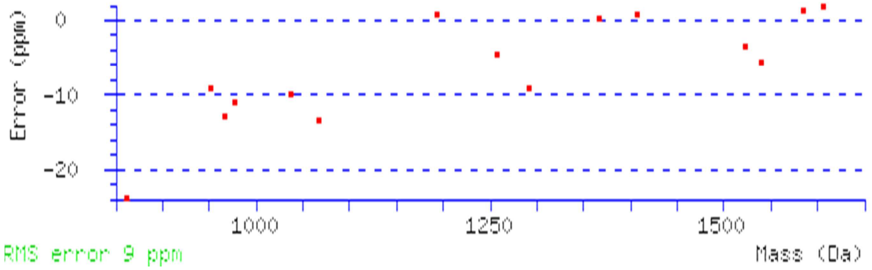

Mascot Search Results for the 29 kDa protein spot

User :
Email :
Search title : SampleSetID: 304, AnalysisID: 3076, MaldiWellID: 36632, SpectrumID: 74705, Path=\CNB_141104\Lola141104 MS\14-120 Rosa su db
Database : Sarcoptes Rosa (1 sequences; 566 residues)
Timestamp : 11 Nov 2014 at 12:13:42 GMT
Top Score : 67 for P00001, Sarcoptes scabiei 15-2-A

Mascot Score Histogram

Protein score is -10*Log(P), where P is the probability that the observed match is a random event.
Protein scores greater than 13 are significant (p<0.05).

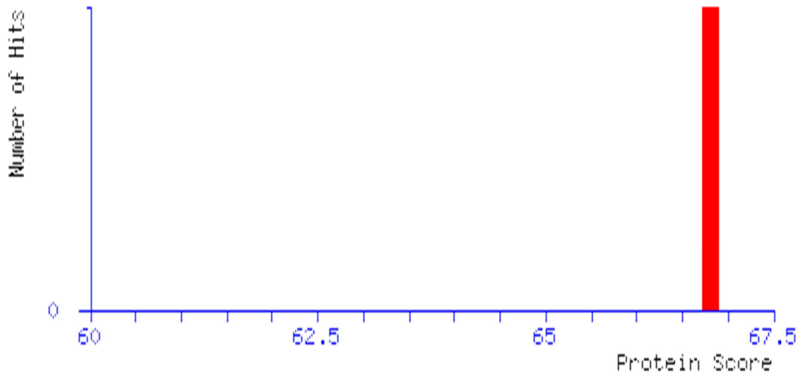

Protein Summary Report

Format As	Protein Summary	Help
	Significance threshold p< 0.05	Max. number of hits 20

Re-Search All

Index

	Accession	Mass	Score	Description
1.	P00001	64953	67	Sarcoptes scabiei 15-2-A

Results List

1. P00001 **Mass:** 64953 **Score:** 67 **Expect:** 2.1e-007 **Matches:** 15

Sarcoptes scabiei 15-2-A

Observed	Mr(expt)	Mr(calc)	ppm	Start		End	Miss	Peptide
863.3875	862.3802	862.4007	-23.76	412	-	418	0	R.LYDGHMK.K
952.4219	951.4146	951.4232	-9.06	466	-	472	0	K.FQNECVR.L
968.4672	967.4599	967.4723	-12.78	505	-	512	0	K.GLHLEDER.G
978.5259	977.5186	977.5294	-11.03	473	-	481	1	R.LAAKDASFR.V
1037.4796	1036.4723	1036.4825	-9.83	62	-	70	0	K.ESISEYGPR.I
1068.5726	1067.5653	1067.5797	-13.49	551	-	559	1	R.SMVHEPKIK.Q
1193.6104	1192.6031	1192.6023	0.72	464	-	472	1	R.LKFQNECVR.L
1258.7209	1257.7136	1257.7193	-4.54	394	-	403	1	K.YVINRVEPIR.V
1291.6788	1290.6715	1290.6833	-9.12	525	-	534	0	K.VNPWQLELHR.E
1366.7078	1365.7005	1365.7001	0.34	505	-	516	1	K.GLHLEDERGNVK.N
1407.7124	1406.7051	1406.7041	0.72	62	-	73	1	K.ESISEYGPRIEK.K
1524.7672	1523.7599	1523.7654	-3.57	491	-	504	0	R.GSVHVAEMEPLDLK.G
1540.7590	1539.7517	1539.7603	-5.56	491	-	504	0	R.GSVHVAEMEPLDLK.G + Oxidation (M)
1586.8153	1585.8080	1585.8060	1.29	1	-	16	1	-.GSPNSARGEVIVSEGK.A
1607.8307	1606.8234	1606.8202	1.99	57	-	70	1	K.VIETKESISEYGPR.I

No match to: 850.4409, 851.3151, 859.5217, 903.5519, 905.4117, 906.4853, 966.4380, 982.5284, 1000.5289, 1012.1270, 1013.1278, 1014.1285, 1034.1184, 1035.1215, 1036.1210, 1050.0966, 1050.5487, 1077.4968, 1117.5150, 1129.6157, 1153.5623, 1163.5876, 1175.5601, 1182.6327, 1184.6357, 1190.5997, 1207.6219, 1223.1538, 1224.1543, 1225.1528, 1229.5223, 1245.1344, 1246.4282, 1296.6621, 1306.6605, 1320.6833, 1336.6720, 1348.7004, 1364.6843, 1388.6844, 1412.1792, 1414.1847, 1420.6364, 1423.6693, 1465.7380, 1546.7543, 1749.9333, 2163.0525, 2185.0447, 2216.9685

Search Parameters

Type of search : Peptide Mass Fingerprint
Enzyme : Trypsin
Fixed modifications : Carbamidomethyl (C)
Variable modifications : Oxidation (M)

```
Mass values            : Monoisotopic
Protein Mass           : Unrestricted
Peptide Mass Tolerance : ± 50 ppm
Peptide Charge State   : 1+
Max Missed Cleavages   : 1
Number of queries      : 65
```
